# FastGroupII: A web-based bioinformatics platform for analyses of large 16S rDNA libraries

**DOI:** 10.1186/1471-2105-7-57

**Published:** 2006-02-07

**Authors:** Yanan Yu, Mya Breitbart, Pat McNairnie, Forest Rohwer

**Affiliations:** 1 Department of Biology, San Diego State University, San Diego, CA, USA; 2 Center for Microbial Sciences, San Diego State University, San Diego, CA, USA

## Abstract

**Background:**

High-throughput sequencing makes it possible to rapidly obtain thousands of 16S rDNA sequences from environmental samples. Bioinformatic tools for the analyses of large 16S rDNA sequence databases are needed to comprehensively describe and compare these datasets.

**Results:**

FastGroupII is a web-based bioinformatics platform to dereplicate large 16S rDNA libraries. FastGroupII provides users with the option of four different dereplication methods, performs rarefaction analysis, and automatically calculates the Shannon-Wiener Index and Chao1. FastGroupII was tested on a set of 16S rDNA sequences from coral-associated *Bacteria*. The different grouping algorithms produced similar, but not identical, results. This suggests that 16S rDNA datasets need to be analyzed in multiple ways when being used for community ecology studies.

**Conclusion:**

FastGroupII is an effective bioinformatics tool for the trimming and dereplication of 16S rDNA sequences. Several standard diversity indices are calculated, and the raw sequences are prepared for downstream analyses.

## Background

Less than 1% of environmental microbes are readily culturable using standard methods [1]. Studies of total microbial diversity must therefore use culture-independent approaches. The breakthrough to these types of studies occurred when Woese et al. [2] proposed the Domains of *Bacteria*, *Archaea*, and *Eucarya *based on small subunit ribosomal DNA sequences (rDNA). Conserved regions within the rDNA genes make it possible to clone directly from environmental samples, allowing uncultured microbial diversity to be surveyed [3-5]. Sequencing 16S rDNAs is now a standard technique for analyzing environmental microbial communities. As the time and costs required for sequencing continue to decrease, researchers are obtaining increasingly large 16S rDNA libraries. Bioinformatic tools for efficiently and accurately analyzing these data are now essential.

Here we present FastGroupII, a web-based platform for the dereplication of large 16S rDNA libraries and estimation of community composition and diversity. Within a few seconds, FastGroupII can trim and dereplicate a library containing thousands of 16S rDNA sequences based on user-defined criteria. This tool provides the user with the option of four different algorithms to group similar sequences together (i.e., to dereplicate sequences). FastGroupII then calculates standard species richness estimators and biodiversity indices. The output from FastGroupII is a FASTA formatted file containing a representative sequence from each user-defined group, which can then be directly input to sequence classification programs.

## Implementation

### Software design and computer hardware configuration

FastGroupII is web-based and accessible at FastGroupII homepage [6]. The software package was developed in Perl5.8 (Open Source Software). The web interface was developed using the CGI module in Perl. FastGroupII currently runs on a DEX (Data Exchange Corporation; Camarillo, California) 200 MHz Pentium4 PC server. The web service is supported using Apache HTTP server (Open Source Software). The source code for FastGroupII is also available at this website.

### Test 16S rDNA library

A library containing bacterial 16S rDNA sequences from four species of corals (*Montastraea franski*, *Diploria strigosa*, *Porites astreoides *and *P. divaricata*) was used to test FastGroupII (sequences from [7] and unpublished data). The library was made by PCR amplifying total community DNA with 27F (5' AGAGTTTGATCMTGGCTCAG 3') and 1492R (5' TACGGYTACCTTGTTACGACTT 3') primers. The products were cloned into pCR4.0-TOPO vector (Invitrogen; Carlsbad, CA), and the inserts were sequenced with the 27F primer. All sequences in the test dataset are unedited single pass reads. The test dataset is available on the User's Guide page of the FastGroupII website.

## Results and discussion

### Overview of FastGroupII online analyses tool

**Figure 1:**
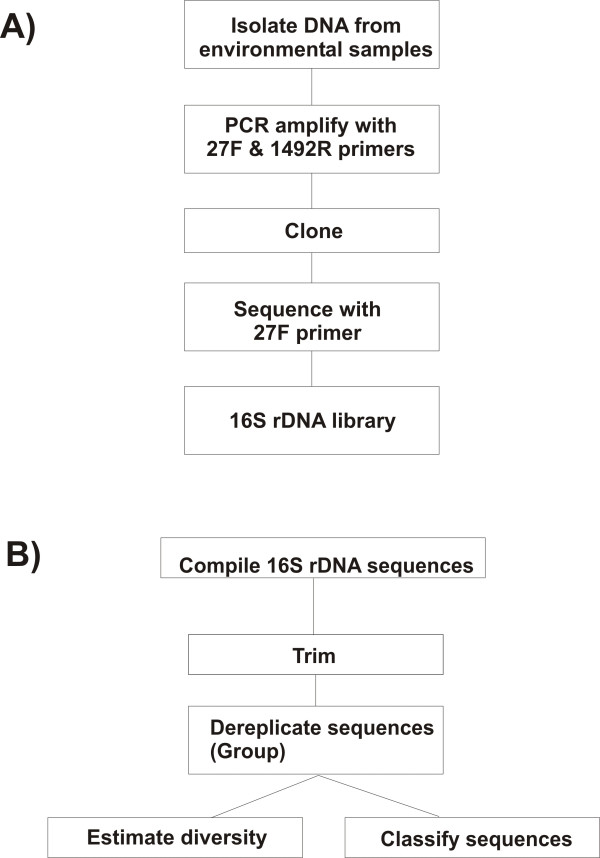
Overview of the process for analyzing microbial communities using FastGroupII. A) Protocol for high-throughput sequencing of environmental microbial communities. B) Protocol for 16S rDNA analyses used in FastGroupII. Sequences are trimmed and dereplicated according to user-specified parameters. FastGroupII can perform rarefaction analysis, and calculate the Chao1 richness estimator and the Shannon-Wiener diversity index. The output from FastGroupII is formatted for submission to sequence classification programs such as BLAST [10] and RDP Classifier [11].

FastGroupII is web-based and accessible at FastGroupII [6]. Figure 1A is an overview of the typical protocol for obtaining 16S rDNA libraries from environmental microbial communities. FastGroupII processes the raw sequence files by trimming, grouping similar sequences together, calculating diversity indices, and preparing an output that is suitable for subsequent sequence classification (Figure 1B). Briefly, sequences are loaded into FastGroupII as a FASTA formatted document and trimmed with user-specified parameters. Closely related sequences are grouped together using one of four available dereplication algorithms. The user can easily change the grouping parameters by which sequences are dereplicated into different ribotypes. FastGroupII then outputs a statistical estimation of richness (Chao1; [8]), a diversity index (Shannon-Wiener Index; [9]), and a text file of dereplicated sequences that can be used for BLAST [10] or analyses with the Ribosomal Database Project (RDP; [11,12]). Rarefaction and rank-abundance curves can also be visualized in graphical format.

### Importing and trimming sequences

**Figure 2:**
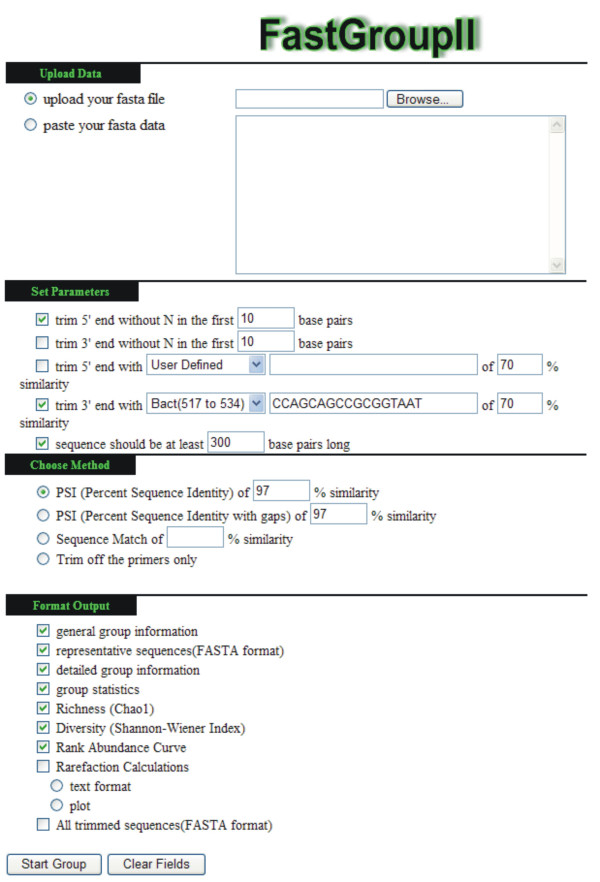
FastGroupII online analyses tool at FastGroupII Tools [6]. A FASTA formatted file containing the raw 16S rDNA sequences is first uploaded or pasted as the input file. The user then specifies the trimming and grouping criteria and selects the desired output. After submission, analysis is performed on the remote server and results are returned to the user on the same web page.

The web interface for FastGroupII is shown in Figure 2. To import sequences into FastGroupII, it is necessary that sequences are precompiled and stored in a FASTA formatted plain text file. A program, named "Converter", for compiling a folder of text files (.seq extensions) into one FASTA file can be downloaded from the FastGroupII website. Sequences can be input to FastGroupII either by pasting into the appropriate window, or by uploading a FASTA file via the browse window.

FastGroupII can trim sequences in two ways: 1) sequences with a certain proportion of ambiguous bases (e.g., "N"s) are removed from the ends, and/or 2) bases 5' or 3' of a user-specified site are removed (e.g., the conserved site in *Bacteria *at position 534). A detailed analysis of different trimming criteria was presented in [13]. Several frequently used conserved 16S rDNA sites of *Bacteria *and *Archaea *are listed in a pull-down menu at the FastGroupII website. To ensure the quality of the sequences for subsequent analyses, a minimum length requirement for the trimmed sequences can also be specified.

### Dereplicating sequences

One main feature of FastGroupII is the dereplication process, in which identical or nearly-identical sequences belonging to the same microbial ribotype are grouped together. FastGroupII incorporates four dereplication algorithms: PSI (Percentage Sequence Identity) [13], PSI with Gaps, Seq-Match [12], and a Tree-parsing method based on ClustalW alignments [14]. With the exception of the Tree-parsing method, similar sequences are grouped together according to the following steps.

Sequences are first trimmed according to the user-specified parameters. After trimming, the first sequence in the library is read into memory and automatically becomes a Representative Sequence. The next sequence (the Query Sequence) is then read into memory and compared to the Representative Sequence. If the Query Sequence is similar to the Representative Sequence, according to the user-specified criteria, it is added to the same group as the first Representative Sequence. If not, the Query Sequence becomes the Representative Sequence of a new group. This process is repeated with the next Query sequence in the dataset being compared to the Representative Sequence of each group until all the sequences in the library have been compared. Specific details of each grouping algorithm available in FastGroupII are described below.

### Percentage sequence identity algorithm

The Percentage Sequence Identity (PSI) algorithm [13] compares each base in the Query Sequence to each base in the Representative Sequence in a pair-wise fashion. The comparison between the Query and Representative Sequence starts at the user-defined end of the sequence (3' end unless sequences are trimmed to a 5' site), and continues sequentially. One match is counted for each position where the two bases being compared are identical; each position where the bases are different is counted as a mismatch. The comparison stops when the last base in the shorter sequence is reached. The PSI is then calculated by dividing the number of matches between the sequences by the number of bases in the shorter sequence. A Query Sequence is included into a previously established group if the PSI between the Query Sequence and Representative Sequence of that group is greater than the user-defined threshold value.

### PSI with gaps algorithm

With the PSI algorithm, insertion or deletion of a single base (i.e., a frameshift) will cause all the subsequent positions to be mismatches. This can lead to a situation where two sequences only differ by a single base, but the frameshift causes the sequences to have an extremely low PSI value, which classifies them into separate groups (see Additional file 1). These single base gaps may be due to true heterogeneity in the sequences, however, it must be cautioned that single base insertions or deletions are common sequencing errors associated with single-pass sequences.

In order to circumvent this error, the PSI with Gaps algorithm was developed. The PSI with Gaps algorithm carries out the comparison in the same manner as the PSI algorithm, with the exception that when a mismatch is recorded during comparison, the PSI with Gaps algorithm attempts to insert a gap into the Query Sequence or the Representative Sequence to make a match. If a match is found after a gap adjustment, the comparison continues from the base after the gap. The PSI with Gaps algorithm implemented in FastGroupII allows a maximum gap adjustment of 2 consecutive, base pairs.

Using the test 16S rDNA library, there were ~23% fewer unique groups obtained using the PSI with Gaps algorithm compared to the PSI method. Visual inspection of these sequences showed that sequencing errors were the most likely explanation for this discrepancy. Therefore, it is suggested that the PSI with Gaps algorithm be used for most datasets. However, single base insertions and deletions can represent true heterogeneity in 16S rDNA groups (e.g., [15]). Studies of micro-heterogeneity require sequencing to a higher coverage, and should be analyzed using a true pair-wise comparison like the PSI algorithm.

### Seq-match algorithm

The Seq-Match algorithm was modified from the Sequence Match function in the RDP project [12] developed at Michigan State University. The Seq-Match method first encodes a sequence into a list of integers by translating each n-oligomer in the sequence into an integer (ranging from zero to 4^n^). Unique integers are then stored in a list that represents the sequence. If an integer in the list of the Query Sequence is also found in that of the Representative Sequence, one match is counted. The Seq-Match score between the Query Sequence and the Representative Sequence is calculated as the number of matching integers divided by the number of integers in the shorter list.

Since the Seq-Match method compares the two lists of integers encoded from the sequences, rather than directly comparing the bases in a pair-wise fashion, a conversion method between the PSI and Seq-Match grouping thresholds was developed. More details of the correlation between PSI and Seq-Match, along with practical examples, can be found in Additional file 1. Briefly, if an oligomer size of *n *is used for encoding the list of unique integers from a sequence, a mismatch in one sequence can result in a maximum of *n *different integers. However when multiple mismatches occur in the comparison, it is not always the case that each of the mismatches causes *n *different integers. For example, when two mismatches are ≥ *n *bases apart, it results in the maximum number of different integers per mismatch on average. But if two contiguous mismatches occur, it results in *n*+1 different integers in total, and thus (*n*+1)/2 different integers for each mismatch on average. The case just described causes the minimal number of different integers per mismatch. Any mismatches located less than *n *bases apart from each other will cause an intermediate number of differences. As a result, there are no accurate criteria for a grouping threshold in the Seq-Match method. Averaging out the maximum and minimum number of differences that can be caused by a mismatch predicts that each mismatch will cause an average of (3*n*+1)/4 different integers.

Assuming that microbes with 16S rDNA ≥ *m*% in PSI are considered the same ribotype, the corresponding percentage identity in the Seq-Match method is calculated as:

O−L×(1−m%)×(3n+14)O     (EQ1)
 MathType@MTEF@5@5@+=feaafiart1ev1aaatCvAUfKttLearuWrP9MDH5MBPbIqV92AaeXatLxBI9gBaebbnrfifHhDYfgasaacH8akY=wiFfYdH8Gipec8Eeeu0xXdbba9frFj0=OqFfea0dXdd9vqai=hGuQ8kuc9pgc9s8qqaq=dirpe0xb9q8qiLsFr0=vr0=vr0dc8meaabaqaciaacaGaaeqabaqabeGadaaakeaadaWcaaqaaiabd+eapjabgkHiTiabdYeamjabgEna0kabcIcaOiabigdaXiabgkHiTiabd2gaTjabcwcaLiabcMcaPiabgEna0kabcIcaOmaalaaabaGaeG4mamJaemOBa4Maey4kaSIaeGymaedabaGaeGinaqdaaiabcMcaPaqaaiabd+eapbaacaWLjaGaaCzcamaabmaabaGaeeyrauKaeeyuaeLaeGymaedacaGLOaGaayzkaaaaaa@47E4@

where *O *is the number of unique integers (oligomers of length *n*); *L *is the length of the shorter sequence; *n *is the length of the oligomer; and *m *is the percentage identity of direct comparison. A simplified version of this relationship is:

1−(1−m%)×(3n+14)A     (EQ2)
 MathType@MTEF@5@5@+=feaafiart1ev1aaatCvAUfKttLearuWrP9MDH5MBPbIqV92AaeXatLxBI9gBaebbnrfifHhDYfgasaacH8akY=wiFfYdH8Gipec8Eeeu0xXdbba9frFj0=OqFfea0dXdd9vqai=hGuQ8kuc9pgc9s8qqaq=dirpe0xb9q8qiLsFr0=vr0=vr0dc8meaabaqaciaacaGaaeqabaqabeGadaaakeaacqaIXaqmcqGHsisldaWcaaqaaiabcIcaOiabigdaXiabgkHiTiabd2gaTjabcwcaLiabcMcaPiabgEna0kabcIcaOmaalaaabaGaeG4mamJaemOBa4Maey4kaSIaeGymaedabaGaeGinaqdaaiabcMcaPaqaaiabdgeabbaacaWLjaGaaCzcamaabmaabaGaeeyrauKaeeyuaeLaeeOmaidacaGLOaGaayzkaaaaaa@4454@

where *A *= *O/L *is the average percentage of unique oligomers divided by the length of the sequence. For the test dataset used in this paper, *A *= 97%. If an oligomer size of 7 is used to encode the sequences (*n *= 7), given 97% pair-wise PSI, a grouping criterion of 83% in the Seq-Match method is calculated correspondingly.

As shown in Table 1, the number of groups obtained in the Seq-Match method is 13% fewer than that obtained using the PSI with Gaps method. Further analysis showed that some sequences which were grouped by a Seq-Match threshold of 83% were only 95%-96% identical using the PSI with Gaps method. The results obtained with this test dataset exemplify the fact that the equations presented above only provide an approximate comparison between the two grouping algorithms.

**Table 1:** Comparison of different grouping algorithms available within FastGroupII and DOTUR. A total of 621 16S rDNA sequences were grouped 20 times using the PSI, PSI with Gaps, and Seq-Match methods. During each separate grouping, Query Sequences were chosen at random to determine if there was any effect of input order. Data from these 20 groupings are shown as the average ± standard deviation. The Tree-parsing and DOTUR methods use global alignments, so randomization was not used. The 3 methods in DOTUR use the PHYLIP distance matrix generated from a global alignment in ClustalW (FN: Furthest Neighbor, NN: Nearest Neighbor, AN: Average Neighbor).

	**PSI**	**PSI with Gaps**	**Seq-Match**	**Tree-parsing**	**DOTUR**		
					
					**FN**	**NN**	**AN**
# of groups	209 ± 2	160 ± 4	140 ± 3	200	132	122	126
Richness (Chao1)	599 ± 27	359 ± 22	281 ± 8	440	249	241	246
Diversity (Shannon-Wiener)	3.98 ± 0.04	3.62 ± 0.10	3.35 ± 0.19	4.5	3.58	3.04	3.07
# of singletons	148 ± 2	99.7 ± 3.2	80.8 ± 1.7	120	72	69	71
# of doubletons	28.2 ± 1.5	25.3 ± 2.7	23.2 ± 0.9	29	22	20	21

### Tree-parsing algorithm

The Tree-parsing algorithm implemented in FastGroupII uses a guide tree obtained from ClustalW [14]. ClustalW is a widely used tool for multiple sequence alignments, but has the disadvantage that it does not automatically group sequences based on user-defined criteria. The Tree-parsing method is fundamentally different than the other grouping methods because it is based on a global alignment algorithm rather than a pair-wise comparison. The link for the Tree-parsing algorithm is located on the main FastGroupII page. First, each sequence in the input list is aligned to each other sequence and a distance matrix reflecting the divergence of each sequence pair is calculated. The scores in the distance matrix are calculated as the number of identities in the best alignment divided by the number of bases compared (gap positions are excluded). Second, a guide tree is built from the distance matrix using the neighbor-joining method. Finally, the sequences are aligned progressively according to the branching order in the guide tree [14].

In the progressive alignment method, the most closely related sequences are aligned first. The guide tree calculated in the second step is built upon the distance matrix and the branch lengths are proportional to the estimated divergence along each branch. The Tree-parsing method implemented in FastGroupII retrieves the branch lengths in the guide tree and uses them to group the closely related nodes together according to the user-specified PSI threshold.

Results from the Tree-parsing method should be similar to the results of a pair-wise alignment, although the similarity scores will vary depending on the substitution matrix specified by the user in the ClustalW alignment. There were 25% more groups obtained from dereplication of the test dataset with the Tree-parsing method than obtained with the PSI with Gaps method. Table 1 shows that this disparity was mainly due to the fact that the number of groups with only one sequence (singletons) and the numbers of groups with only two sequences (doublets) from the Tree-parsing method outnumbered the PSI with Gaps method. This is due to the fact that the substitution matrix used for the ClustalW pair-wise alignment weighs different base substitutions or gaps differently, while the PSI with Gaps method regards all cases of mismatches/gaps as the same.

### Comparison of the four dereplication algorithms

The PSI method is the fastest method for sequence dereplication, however, single base insertions or deletions can cause overestimations of richness and diversity. The PSI with Gaps and Seq-Match methods have a higher tolerance to insertions or deletions. However, it is impossible to directly correlate PSI and Seq-Match similarity thresholds. In addition, the Seq-Match method can easily be affected by factors such as the sequence length, or a given *n*-oligomer being present more than once within a sequence. The Tree-parsing method was more than 700 times slower than the other 3 methods (Table 2) for the analyses of ~600 sequences. Most of the computation time was consumed by the dynamic programming alignment method used in ClustalW [14]. The PSI with Gaps method can reduce the error caused by sequencing insertions or deletions while maintaining a fast computing performance, and is therefore recommended for most routine analyses.

**Table 2:** Speed of the 4 grouping methods in FastGroupII, and a comparison with FastGroup 1.0. The time in seconds was determined by trimming and grouping the 16S rDNA test dataset found on the FastGroupII website. A total of 621 sequences were dereplicated. A percentage sequence identity of 97% was used to group similar sequences in the PSI, PSI with Gaps and Tree-parsing method. A percentage sequence identity of 83% was used in the Seq-Match method.

**Method**	**Time (s)**
PSI	2
PSI with Gaps	5
Seq-Match	10
Tree-parsing	7152 (ClustalW) + 0.1 (tree-parsing time)
FastGroup 1.0	360

### Output from fastGroupII

After trimming and dereplication, FastGroupII generates a FASTA formatted file containing a representative sequence for each group. The number of sequences in each group (group statistics) is also produced as a list. The output from FastGroupII is ready for further analyses using other tools (e.g., BLAST [10] or Classifier in RDP [11]) to reveal specific information of interest.

### Calculating richness estimators and biodiversity indices

Sequencing 16S rDNA has extended the study of microbial biodiversity to new levels. It is still impractical, however, to exhaustively sample a whole microbial community. Statistical approaches that are traditionally used to study macroorganisms can also be applied to microbial communities [16]. These approaches can make predictions about total community diversity based on a subsample of sequences (reviewed in [17]).

FastGroupII implements several of these estimators including Chao1 [8,18], the Shannon-Wiener Index [9] and rarefaction analysis [19,20]. Chao1 (EQ3) is a simple nonparametric estimator of the minimum richness (i.e., number of ribotypes) in a sample. In FastGroupII, a ribotype is defined as sequences that are grouped together because they are above the user-specified threshold for similarity. Chao1 is based on the number of rare ribotypes (singletons and doublets) within a sample.

Schao1=Sobs+n12/(2n2)     (EQ3)
 MathType@MTEF@5@5@+=feaafiart1ev1aaatCvAUfKttLearuWrP9MDH5MBPbIqV92AaeXatLxBI9gBaebbnrfifHhDYfgasaacH8akY=wiFfYdH8Gipec8Eeeu0xXdbba9frFj0=OqFfea0dXdd9vqai=hGuQ8kuc9pgc9s8qqaq=dirpe0xb9q8qiLsFr0=vr0=vr0dc8meaabaqaciaacaGaaeqabaqabeGadaaakeaacqWGtbWudaWgaaWcbaGaem4yamMaemiAaGMaemyyaeMaem4Ba8MaeGymaedabeaakiabg2da9iabdofatnaaBaaaleaacqWGVbWBcqWGIbGycqWGZbWCaeqaaOGaey4kaSIaemOBa42aa0baaSqaaiabigdaXaqaaiabikdaYaaakiabc+caViabcIcaOiabikdaYiabd6gaUnaaBaaaleaacqaIYaGmaeqaaOGaeiykaKIaaCzcaiaaxMaadaqadaqaaiabbweafjabbgfarjabiodaZaGaayjkaiaawMcaaaaa@4B5B@

where *S*_*obs *_is the observed number of ribotypes; and *n*_1 _and *n*_2 _are the number of ribotypes observed either once or twice respectively. The Chao1 prediction will exceed the number of observed ribotypes by an amount that is determined by the number of singletons.

Rarefaction measurement (EQ4) corrects for the effects of sample size on richness predictions by scaling all the samples down to the same size [14,15]. In rarefaction analysis, the information provided by all the ribotypes sampled is used to estimate the richness of a smaller sample, allowing for direct comparisons to be made between communities of different sizes.

E(Sn)=∑i=1s[1−(N−Nin)/(Nn)]     (EQ4)
 MathType@MTEF@5@5@+=feaafiart1ev1aaatCvAUfKttLearuWrP9MDH5MBPbIqV92AaeXatLxBI9gBaebbnrfifHhDYfgasaacH8akY=wiFfYdH8Gipec8Eeeu0xXdbba9frFj0=OqFfea0dXdd9vqai=hGuQ8kuc9pgc9s8qqaq=dirpe0xb9q8qiLsFr0=vr0=vr0dc8meaabaqaciaacaGaaeqabaqabeGadaaakeaacqWGfbqrcqGGOaakcqWGtbWudaWgaaWcbaGaemOBa4gabeaakiabcMcaPiabg2da9maaqahabaWaamWaaeaacqaIXaqmcqGHsislcqGGOaakcqWGobGtcqGHsisldaWcaaqaaiabd6eaonaaBaaaleaacqWGPbqAaeqaaaGcbaGaemOBa4gaaiabcMcaPiabc+caViabcIcaOmaalaaabaGaemOta4eabaGaemOBa4gaaiabcMcaPaGaay5waiaaw2faaaWcbaGaemyAaKMaeyypa0JaeGymaedabaGaem4CamhaniabggHiLdGccaWLjaGaaCzcamaabmaabaGaeeyrauKaeeyuaeLaeGinaqdacaGLOaGaayzkaaaaaa@513A@

The Shannon-Wiener Index (EQ5) is a nonparametric diversity index that combines estimates of richness (the total number of ribotypes) and evenness (the relative abundance of each ribotype):

*H*' = -∑[*P*_*i*_(ln *P*_*i*_)]     (EQ5)

where *P*_*i *_is the proportion of individuals found in the *i*th ribotype of the community. The Shannon-Wiener Index can be used as an overall indicator of the level of diversity in a sample.

FastGroupII can also display standard rank-abundance curves. In these plots, ribotypes are plotted from most to least abundant along the x-axis, with their abundances displayed on the y-axis. Rank-abundance curves reveal differences in patterns of richness and evenness between samples. In addition, the shape of the rank-abundance curve can be used to determine which species-abundance model best fits the data (e.g., power law, logarithmic, lognormal, etc...). Determining the shape of rank-abundance curves for microbial communities has important implications for predictions of the total number of microbial ribotypes on the planet [21].

### Effect of sequence order on grouping

One bias that can be caused in the PSI, PSI with Gaps and Seq-Match grouping methods results from the selection of the Representative Sequences. In these three grouping algorithms, the first sequence put into a new group is designated as the Representative Sequence. This random selection process might lead to differences in the results of dereplication.

The effects of sequence order in the input file were evaluated by dereplicating the sample dataset 20 times using each method. For each trial, the sequences in the dataset were read into the program in a random order. By doing this, a different sequence was selected as the Representative Sequence of a new group each time. The average value and standard deviation of the number of groups, species richness (Chao1) and diversity (Shannon-Wiener Index) were then calculated. As shown in Table 1, the deviation of each value was less than 3% for the total number of groups using any of the dereplication methods. The deviation was less than 7% for the Shannon-Wiener Index and prediction of richness using Chao1. These results indicated that the method of using the first sequence put into a group as the Representative Sequence only has a minor effect on community composition predictions.

### Comparison of fastGroupII with other methods

FastGroupII was compared with two other available programs (FastGroup 1.0 and DOTUR). FastGroup 1.0 [13] is a Java program that trims and dereplicates sequences based on user-defined criteria. DOTUR [22] is a Windows and Unix-based program that dereplicates 16S rDNA libraries using a distance matrix as input (e.g., from ARB [23]). In addition, DOTUR calculates various richness and diversity indices.

For comparison, FastGroup 1.0 [13] was downloaded and installed on the same server as FastGroupII. FastGroupII was over 100 times faster than FastGroup 1.0 for analyses on the test dataset presented here. The disparities in the execution time can be caused by the implementation details and the performance differences of the two programming languages. The web-based interface of FastGroupII makes it more accessible than the previous Java version.

DOTUR [22] is another publicly available dereplication program. Unlike the FastGroup programs, DOTUR starts with a distance matrix exported from an alignment program like ARB [23] or ClustalW [14]. The same test dataset was used to compare the grouping results from DOTUR to those obtained using FastGroupII. For consistency, the sequences in the test dataset were first trimmed in the same manner as they were in FastGroupII. The trimmed sequences were then aligned using ClustalW with the default alignment parameters. The PHYLIP [24] distance matrix was exported from the global alignment and input to DOTUR using the default parameters. The operational taxonomic units (i.e., groups) defined with 97% similarity were then read from the relevant files generated by DOTUR.

**Figure 3:**
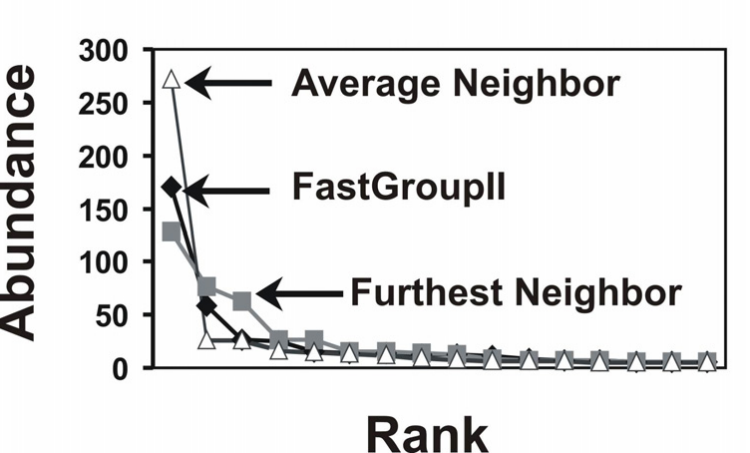
Rank-abundance curves predicted from the test dataset using FastGroupII and methods in DOTUR. The curves reveal similar grouping patterns predicted using the different methods. For clarity, the tails of singletons were excluded from the figure.

As shown in Table 1, the total number of unique groups obtained using DOTUR was fewer than that obtained using the PSI with Gaps algorithm in FastGroupII. A closer examination of the grouping results showed that the distribution pattern differed in the number of individuals in the most abundant group and the number of singletons (Figure 3). Some of the singletons found in the PSI with Gaps algorithm were combined into larger groups by DOTUR. The random selection of the Representative Sequence in the PSI with Gaps algorithm is one of the factors accounting for the variation. However, the differences in the results are most likely due to variation in the substitution matrix used in the alignment, or differences in the classification of groups based on the guide tree.

## Conclusion

FastGroupII is a web-based bioinformatic tool for rapidly trimming and dereplicating 16S rDNA sequences. The user can choose between four different algorithms for dereplicating sequences. FastGroupII allows investigators to determine information about community structure and diversity from 16S rDNA sequence data, and easily format the data for other analyses (e.g., BLAST and ARB).

## Availability

The FastGroupII program is available at FastGroupII [6].

## Abbreviations

rDNA – ribosomal DNA sequences, RDP – Ribosomal Database Project, PSI – Percentage Sequence Identity

## Authors' contributions

YY wrote the PERL scripts, designed the webpage, and performed some of the lab work. MB performed most of the lab work and tested the FastGroupII program. PM set up the FastGroupII server and developed the webpage. FR conceived of the study, coordinated the lab, computer work, and manuscript preparation. All authors read and approved the final manuscript.

## Supplementary Material

Additional file 1 Examples of different dereplication algorithms. Click here for file
