# Supplementary material for: FastGroupII: A web-based bioinformatics platform for analyses of large 16S rDNA libraries
Source: BMC Bioinformatics. 2006 Feb 7;7:57. doi: 10.1186/1471-2105-7-57 (PMC1386709; doi:10.1186/1471-2105-7-57)
Supplement: Additional file 1 — Examples of different dereplication algorithms. [file 1471-2105-7-57-S1.doc]

**FastGroupII: A web-based bioinformatics platform for analyses of large 16S rDNA libraries**

Yanan Yu, Mya Breitbart, Pat McNairnie, and Forest Rohwer

# Additional File 1: Examples of different dereplication algorithms.

# PSI vs. PSI with Gaps

PSI and PSI with Gaps are both pair-wise comparisons between two sequences. With the PSI algorithm, insertion or deletion of a single base will cause a frameshift, making all subsequent positions mismatches. Since single base insertions and deletions are common sequencing errors, a method that can circumvent this error is needed.

Example:

Sequence 1: AGCTTACGTCATGCAT…

Sequence 2: AGCTTATCGTCATGCCT…

In the two sequences listed above, there is a one base insertion in the second sequence. Assume that each sequence is 100 bases long.

Using the PSI method, these methods are only 6% identical (because the first six bases are identical, and every position after that is a mismatch). These sequences would therefore be classified into different groups.

Using the PSI with Gaps method, FastGroupII would insert a one base gap into Sequence 1, which would make all subsequent positions align. Therefore, the sequences would be 99% identical (except for the 1 gap), and would be classified into the same group.

**PSI vs. Seq-Match**

Sequence 1: AGCTTACCGTCATGCCT…

Sequence 2: AGCTTATCGTCATGCCT…

In the two sequences listed above, there is only one different base. Assume each sequence is 100 bases long.

Using the PSI method, these sequences are 99% identical (because 99 out of the 100 bases are identical; 99/100 = 0.99)

The Seq-Match method (using an oligomer size *n*=7) generates the following lists of unique integers for these sequences.

Sequence 1: Sequence 2:

1) AGCTTAC AGCTTAT

2) GCTTACC GCTTATC

3) CTTACCG CTTATCG

4) TTACCGT TTATCGT

5) TACCGTC TATCGTC

6) ACCGTCA ATCGTCA

7) CCGTCAT TCGTCAT

8) CGTCATG CGTCATG

9) GTCATGC GTCATGC

10) TCATGCC TCATGCC

All subsequent integers are the same, since the rest of the bases are identical. Since the integers that are encoded are overlapping (that is, oligomers of length 7 bases, started every base), 7 of the 94 integers generated for each sequence are different. The Seq-Match score is therefore only 93% (because 87 out of the 94 integers generated are identical; 87/94 = 0.93). In this example, the single different base results in the maximum number of different integers (*n*).

Now consider a slight modification on the above example:

Sequence 1: AGCTTACAGTCATGCCT…

Sequence 2: AGCTTATCGTCATGCCT…

In the two sequences listed above, there are two different bases, which occur directly next to each other. Assume each sequence is 100 bases long.

Using the PSI method, these sequences are 98% identical (because 98 out of the 100 bases are identical; 98/100 = 0.98)

The Seq-Match method (using an oligomer size *n*=7) generates the following lists of unique integers for these sequences.

Sequence 1: Sequence 2:

1) AGCTTAC AGCTTAT

2) GCTTACA GCTTATC

3) CTTACAG CTTATCG

4) TTACAGT TTATCGT

5) TACAGTC TATCGTC

6) ACAGTCA ATCGTCA

7) CAGTCAT TCGTCAT

8) AGTCATG CGTCATG

9) GTCATGC GTCATGC

10) TCATGCC TCATGCC

11) CATGCCT CATGCCT

All subsequent integers are the same, since the rest of the bases are identical. Since the integers that are encoded are overlapping (that is, oligomers of 7, started every base), 8 of the 94 integers generated for each sequence are different. The Seq-Match score is therefore 91% (because 86 out of the 94 integers generated are identical; 86/94 = 0.91). In this example, the single different base results in the minimum number of different integers (*n*).

Consider a slightly modified version of the example above, where two different bases are separated by two identical bases:

Sequence 1: AGCTTACCGACATGCCT…

Sequence 2: AGCTTATCGTCATGCCT…

In the two sequences listed above, there are only two different bases. Assume each sequence is 100 bases long.

Using the PSI method, these sequences are 98% identical (because 98 out of the 100 bases are identical; 98/100 = 0.98)

The Seq-Match method (using an oligomer size *n*=7) generates the following lists of unique integers for these sequences.

Sequence 1: Sequence 2:

1) AGCTTAC AGCTTAT

2) GCTTACC GCTTATC

3) CTTACCG CTTATCG

4) TTACCGA TTATCGT

5) TACCGAC TATCGTC

6) ACCGACA ATCGTCA

7) CCGACAT TCGTCAT

8) CGACATG CGTCATG

9) GACATGC GTCATGC

10) ACATGCC TCATGCC

11) CATGCCT CATGCCT

All subsequent integers are the same, since the rest of the bases are identical. Since the integers that are encoded are overlapping (that is, oligomers of 7, started every base), 10 of the 94 integers generated for each sequence are different. The Seq-Match score is therefore only 89% (because 84 out of the 94 integers generated are identical; 89/94 = 0.89). In this example, each of the single different base results in less than the maximum number of different integers.

Similarly, if the sequences differed by two bases, but these bases were separated by more than *n* bases, each different base would result in the maximum number of different integers and there would be 14 different integers generated. This would make the Seq-Match score 85%, compared to a PSI score of 98%.

Therefore, having a PSI score of 98% for a sequence of 100 bp long could correspond to a Seq-Match score (with *n*=7) ranging 85% to 91%.
